# 1,3-Butanediol Administration Increases β-Hydroxybutyrate Plasma Levels and Affects Redox Homeostasis, Endoplasmic Reticulum Stress, and Adipokine Production in Rat Gonadal Adipose Tissue

**DOI:** 10.3390/antiox12071471

**Published:** 2023-07-22

**Authors:** Giuliana Panico, Gianluca Fasciolo, Vincenzo Migliaccio, Rita De Matteis, Lillà Lionetti, Gaetana Napolitano, Claudio Agnisola, Paola Venditti, Assunta Lombardi

**Affiliations:** 1 Department of Biology, University of Naples Federico II, Complesso Monte Sant’Angelo Via Cintia 26, 80126 Napoli, Italy; giuliana.panico@unina.it (G.P.); agnisola@unina.it (C.A.); paola.venditti@unina.it (P.V.); 2 Department of Chemistry and Biology “A. Zambelli”, University of Salerno, Via Giovanni Paolo II, 132, 84084 Fisciano, Italy; vmigliaccio@unisa.it (V.M.); llionetti@unisa.it (L.L.); 3 Department of Biomolecular Sciences, University of Urbino Carlo Bo, 61029 Urbino, Italy; rita.dematteis@uniurb.it; 4 Department of Science and Technology, Parthenope University of Naples, 80143 Naples, Italy; gaetana.napolitano@uniparthenope.it

**Keywords:** ketone bodies, ROS, antioxidant enzymes, Nrf2, endoplasmic reticulum stress, adipokines, mitochondrial respiratory complexes

## Abstract

Ketone bodies (KBs) are an alternative energy source under starvation and play multiple roles as signaling molecules regulating energy and metabolic homeostasis. The mechanism by which KBs influence visceral white adipose tissue physiology is only partially known, and our study aimed to shed light on the effects they exert on such tissue. To this aim, we administered 1,3-butanediol (BD) to rats since it rapidly enhances β-hydroxybutyrate serum levels, and we evaluated the effect it induces within 3 h or after 14 days of treatment. After 14 days of treatment, rats showed a decrease in body weight gain, energy intake, gonadal-WAT (gWAT) weight, and adipocyte size compared to the control. BD exerted a pronounced antioxidant effect and directed redox homeostasis toward reductive stress, already evident within 3 h after its administration. BD lowered tissue ROS levels and oxidative damage to lipids and proteins and enhanced tissue soluble and enzymatic antioxidant capacity as well as nuclear erythroid factor-2 protein levels. BD also reduced specific mitochondrial maximal oxidative capacity and induced endoplasmic reticulum stress as well as interrelated processes, leading to changes in the level of adipokines/cytokines involved in inflammation, macrophage infiltration into gWAT, adipocyte differentiation, and lipolysis.

## 1. Introduction

Ketogenesis is a metabolic process leading to the production of ketone bodies (KBs), namely beta-hydroxybutyrate (β-OHB), acetoacetate (AcAc), and acetone. KBs are produced in the liver hepatocytes when excessive fatty acid oxidation and acetyl coenzyme A (acetyl-CoA) production occur [1]. Hepatocytes do not express enzymes that allow the use of KBs as metabolic substrates; KBs are released into the bloodstream and are sent to extrahepatic tissues, where, through ketolysis, they are converted to acetyl-CoA. The latter enters the Krebs cycle to produce energy [1,2].

In addition to being energy molecules, KBs play “non-canonical” roles in the control of energy homeostasis by acting as signal molecules [1,3,4] and by exerting different cellular effects through changes transduced by epigenetic, post-translational, and cell surface signaling mechanisms. In particular, KBs promote proteins post-translational modifications in histone proteins and the inhibition of class I histone deacetylases (HDACI) [5], thus modulating gene transcription. Both β-OHB and AcAc modulate signaling through G protein-coupled receptors (GPRs), with GPR41 and GPR109 specific for β-OHB [6,7] and GPR43 specific for AcAc [8].

Numerous studies have highlighted the clinical benefits of ketosis in various diseases and neurological and metabolic disorders, due to KBs’ cytoprotective actions, actuated by stimulating mitochondrial biogenesis, mitochondrial antioxidant enzymatic defense systems, and preventing lipid and protein oxidative damages [9]. In this context, ketosis, induced by ketogenic diets or by administration of ketone esters, has been used for the treatment of some forms of epilepsy [10], and nowadays, it appears to play a neuroprotective role in neurodegenerative diseases, such as Parkinson’s and Alzheimer’s diseases [11]. Furthermore, KBs can counteract inflammation and non-alcohol-dependent liver disorders and appear to have cardioprotective effects as well [9]. More studies indicate that white adipose tissue (WAT) is a target of KBs signaling [7,12,13], but the effects they induce on WAT physiology are only partially known.

WAT accumulates lipids to redistribute during fasting to other tissues and organs [14] and actively regulates lipid and carbohydrate metabolism in various organs and tissues by secreting multiple biologically active molecules called adipokines. Some adipokines exert local autocrine and paracrine actions, modulating the migration of the immune cell into the tissue, adipogenesis, and adipocyte metabolism [15]. Other adipokines are secreted in the blood, thus allowing the adipocytes to play significant roles in feedback mechanisms at the systemic level, such as the control of energy balance, the regulation of eating behavior, caloric intake, glucose availability, and energy expenditure. Furthermore, other adipokines are released by WAT in response to stressful conditions or obesity, mainly visceral, and are involved in the onset of insulin resistance, metabolic syndrome, and its complications [15].

The function of WAT is intricately linked with redox changes, and lipogenesis and adipogenesis are controlled by the redox shift [16]. In addition, adipocyte functionality is profoundly regulated by the endoplasmic reticulum (ER), which is crucial for lipid synthesis and droplet formation, protein folding and secretion, and Ca^2+^ homeostasis. Indeed, alterations in the ER functionality have been reported to affect WAT lipolysis and inflammation [17,18,19]. The protein folding process within ER has a high energy cost; it is profoundly dependent on redox homeostasis and is strictly linked to mitochondrial functionality. The ER lumen has a slightly oxidizing environment that is functional for the formation of disulfide bonds in structural, membrane, and secretory proteins. Variations of redox conditions in ER lumen, towards either oxidizing or reducing direction, can disrupt the protein folding mechanism, thus enhancing the production of misfolded proteins and activating the unfolded protein response (UPR^ER^) and ER stress [20,21]. When under stress, ER relays calcium signals to mitochondria, which activate substrate oxidation and ROS production, thus leading to mitochondrial dysfunction and establishing a vicious cycle [22].

Given the ability of KBs to influence the redox state and the need for an adequate redox state for WAT physiology, here we studied the effects caused by the enhancement of KBs plasma levels on WAT functionality. Throughout the study, we focused our attention on visceral WAT, in particular on the large gonadal WAT depot (gWAT), as it is of particular concern because it is a key player in metabolic derangements much more than subcutaneous WAT [23,24,25]. Our goal was to shed light on the direct effects induced by KBs, regardless of the multiple pleiotropic effects that occur under physiological conditions in which their levels increase, such as fasting, physical exercise, or following the administration of a ketogenic diet.

Furthermore, 1,3-butanediol (BD) is a di-alcohol that is readily oxidized in liver hepatocytes yielding a rapid enhancement in β-OHB concentration; in particular, in rodents, BD in vivo administration has been used as a strategy to provoke “exogenous” ketosis since it was demonstrated to produce a rapid dose-dependent elevation of β-HOB serum levels, thus providing an alternative method to elevate β-OHB in place of nutrient deprivation [26,27].

In the present work, we administered in vivo BD to rats for 14 days to provoke “exogenous” ketosis. Since β-HOB has been reported to reduce food assumption [28,29], we also evaluated the rapid effect induced by BD within 3 h from its in vivo administration to avoid the confounding effect of reduction of energy intake.

We focused on gWAT antioxidant capacity, ER stress, mitochondrial functionality, and lipolysis. In addition, we evaluated changes in tissue morphology and adipokines production, following 14 days of adaptation of the tissue to elevated β-OHB levels.

## 2. Materials and Methods

### 2.1. Animals

Male Wistar rats of about 270 g were housed one per cage in a temperature-controlled room maintained at 22 ± 2 °C under a 12 h dark/light cycle.

Three groups of animals were used: the first group consisted of control animals receiving a single i.p. administration of physiological solution at the beginning of the treatment (named C), the second group consisted of animals receiving a single i.p. administration of BD (2.5 mol/100 g b.w.) and euthanized 3 h from the i.p. administration (named BD 3 h), and the third group received a single i.p. administration of BD (2.5 mol/100 g b.w.) followed by the oral administration in drinking water (10%) for 14 days (named BD 14 d). All animals were fed with a standard diet ad libitum (4RF21, Mucedola, Italy) and had free access to water or BD water solution.

Drinking BD solution was prepared and replaced daily. Food and BD solution intake was monitored daily, while animal body weight was monitored three times a week. BD treatment did not affect fluid intakes, being about 24 mL/day for all the groups. The energy of BD was taken into account for the evaluation of the energy intake of the rats.

Β-OHB levels were monitored both in control and in KBs treated rats, after 3 h from either the in vivo administration of BD and vehicle or after 14 days of treatment, to assess the efficacy of the treatments. Glucose levels were detected after 12 days of treatments after a brief period of food removal (5 h).

Venus blood samples were obtained from a small cut on the tail, and D β-OHB and glucose were detected using a ketone body meter (Glucomen Areo β-ketone sensor).

At the end of the treatment, the rats were anesthetized using an i.p. injection of sodium Tiopental (40 mg/kg b.w) and euthanized by decapitation. gWAT was excised, weighed, and immediately processed or frozen in liquid nitrogen and stored at −80 °C for later analysis.

This study was carried out under the recommendations in the EU Directive 2010/63 for the Care and Use of Laboratory Animals. All animal protocols were approved by the Committee on the Ethics of Animal Experiments of the University of Naples Federico II and the Italian Minister of Health (Authorization n° 776/2021-PR.). Every effort was made to minimize animal pain and suffering.

### 2.2. Histological Analysis

Samples of gWAT pads were fixed through immersion in 4% (*v*/*v*) formaldehyde in 0.1 M phosphate buffer (overnight, 4 °C). The samples were dehydrated in ethanol, cleared, and embedded in paraffin blocks. The tissues were cut into serial 6 µm thick sections and hematoxylin-eosin staining was used for morphological examination. Sections were viewed with a Nikon Eclipse 80i light microscope (Nikon Instruments, Milan, Italy) at 20× magnification. Images were obtained with a Sony DS-5M camera connected to an ACT-2U image analyzer.

### 2.3. Lipolysis

Lipolysis was evaluated by detecting the diffusion of glycerol from gWAT to the medium [30]. First, 100 mg of gWAT was incubated for 1 h at 37 °C in 500 μL of Krebs Ringer buffer (12 mM HEPES, 121 mM NaCl, 4.9 mM KCl, 1.2 mM MgSO_4_, 0.33 mM CaCl_2_) supplemented with 2% FA-free bovine serum albumin (BSA) and 0.1% glucose. Parallel incubations, performed in the absence and in the presence of 10 µM isoproterenol, were executed in order to detect basal and isoproterenol-stimulated lipolysis, respectively. During incubations, samples were under continuous slight shaking and were gassed with 95% O_2_–5% CO_2_. At the end of the incubation period, an aliquot of the medium was used for the analysis of glycerol content. A commercially available glycerol reagent kit (Merck Life Science S.r.l., Milano, Italy) was used. The absorbance-based enzyme assay kit was converted to fluorescence-based detection through the inclusion of the H_2_O_2_-sensitive dye Amplex UltraRed, following Clark et al. [31].

### 2.4. Western Blot

First, 100 mg of gWAT was homogenized in 300 µL RIPA buffer (150 mM NaCl, 1.0% Triton X-100, 0.5% sodium deoxycholate, 0.1% SDS, 50 mM Tris, pH 8.0) supplemented with a broad-range antiprotease cocktail (Merck, Life Science S.r.l., Milano, Italy). Homogenates were left on ice for 1 h and then centrifuged at 17,000× *g* for 30 min at 4 °C; the resulting supernatants were collected.

Thereafter, 25 μg protein from gWAT lysates was separated by SDS-PAGE and transferred to nitrocellulose membranes. Membranes were blocked with Tris-buffered saline (TBS) containing 0.1% Tween and 5% nonfat dry milk and were subsequently incubated overnight with the appropriate primary antibodies of interest. The second day, membranes were washed three times in TBS-Tween and then were incubated with secondary horseradish peroxidase coupled antibody and processed for enhanced chemiluminescence detection using Excellent Chemiluminescent Substrate Kit (Elabscience, Houston, TX, USA).

The following primary antibodies have been used throughout the study: anti-superoxide dismutase 2 (SOD-2) (ab 13533; abcam, Cambridge, UK), anti-catalase (CAT) (ab 16731 abcam, Cambridge, UK), cocktail of antibody used to detect CI-NDUF88, CII-SDHB, CIII-UQCRC2, CIV-MTCO1 and CV-ATP VA subunits (oxophos ab110413, abcam), anti eIF2-α (L57A5, Cell Signaling, Beverly, MA, USA), anti (ser 51) eIF2alpha phosphorylated form (D9G8, Cell Signaling), anti-Calnexin (ab22595 abcam, Cambridge, UK), anti peroxiredoxin-3 (PRDX-3) (ab 73349 abcam, Cambridge, UK), anti 78-kDa glucose-regulated protein (GRP78-BiP) (ab 108613 abcam, Cambridge, UK), anti glyceraldehyde phosphate dehydrogenase (GAPDH) (3E8AD9 Invitrogen, Monza, Italy), nuclear factor erythroid 2-related factor 2 (Nrf-2) (E-AB-32280 ElabScience, Houston, TX, USA), anti-Tumor Necrosis Factor Alpha (TNF α) (ab1793, abcam, Cambridge, UK). Primary antibodies were diluted 1:1000, with the exception of anti (ser 51) eIF2α phosphorylated form and cocktail of antibody oxophos that were diluted 1:250.

Protein bands were quantified through densitometric analysis (Image J software 1.52t) and normalized based on loading controls GAPDH.

### 2.5. Determination of gWAT ROS Levels, Lipid and Protein Oxidative Damages, and Total Antioxidant Capacities

gWAT ROS levels were measured following the ROS-induced conversion of 2′,7′-dichlorodihydrofluorescin diacetate (DCFH-DA) in the fluorescent dichlorofluorescein (DCF) [32]. Briefly, aliquots of homogenate (25 µg proteins) were incubated in 200 μL of monobasic phosphate buffer 0.1 M, pH 7.4 supplemented with 10 µM DCFH-DA. Then, FeCl_3_ was added (final concentration 100 µM), and the mixture was incubated for 30 min. The conversion of DCFH-DA to the fluorescent product DCF was measured using a microplate reader (Tecan Infinite 200 pro plate reader) with excitation and emission wavelengths of 485 and 530 nm, respectively. The conversion of DCFH to DCF in the absence of homogenate was detected to evaluate the background. A standard curve of DCF has been used in order to calculate the pmol DCF formed.

Lipid and protein oxidative damages were determined in gWAT by evaluating the lipid hydroperoxides levels and protein-bound carbonyls (CO) as reported elsewhere [33,34]

The total antioxidant capacity of gWAT was determined by following the decolorization, at 734 nm, of the mono-cation radical of 2,2′-azinobis-3-ethylbenzothiazoline-6-sulfonic acid (ABTS^•+^), produced by oxidation by 245 mM potassium persulfate of 7 mM ABTS, due to 0.01 mg of gWAT proteins [35]. The calibration curve was obtained using a stock solution of 3,5-Di-tert-4-butylhydroxytoluene (BHT). Total antioxidant capacity was expressed as BHT Equivalents∙ mg^−1^ protein.

### 2.6. Adipokines Content Analysis

A Proteome Profiler rat Adipokine Array kit (R&D Systems, Minneapolis, MN, USA) has been used for the parallel determination of the relative levels of selected Adipokines following the manufacturer’s indication.

### 2.7. Statistical Analysis

Data were analyzed with the GraphPad Prism 6.02 (San Diego, CA, USA) software system. One-way analysis of variance method (ANOVA), followed by the “Tukey correction for Multiple Comparison” or Student’ *t*-test were used to establish the statistical significance of the differences between experimental groups. Differences were considered statistically significant at *p* < 0.05.

## 3. Results

### 3.1. BD Administration Reduces Epididymal gWAT Mass and Adipocyte Size and Promotes Lipolysis

**Figure 1:**
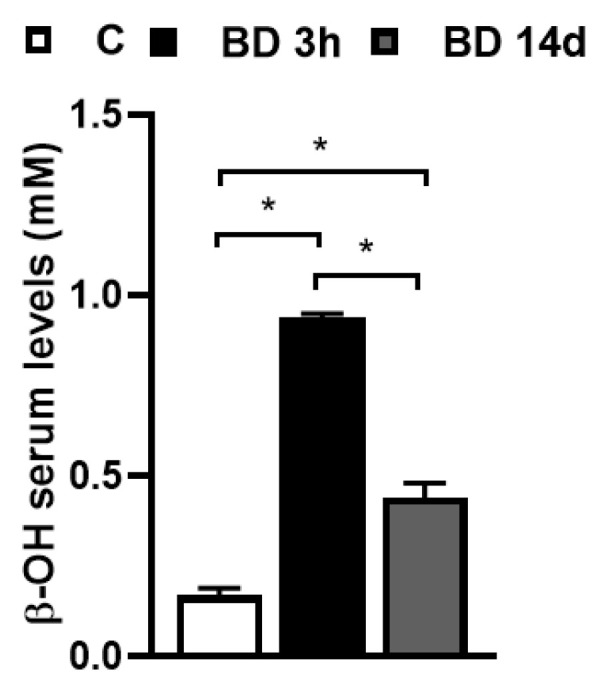
Effect of BD treatment on (D) β-OHB serum levels, detected after 3 h from BD i.p administration (BD 3 h) and after 14 days of oral administration (BD 14 d). Values represent the mean ± SE of 10 different animals. * *p* < 0.0001.

The administration of BD was effective in inducing ketosis since it induces a significant increase in serum (D) β-OHB levels. Three hours after BD i.p. administration (BD 3 h), β-OHB serum levels reached values 5.5-fold higher than the control, and after 14 days of treatment with BD added to drinking water (BD 14 d), the values were about 2.6-fold higher than the control (Figure 1). At the end of the 14 days of treatment, BD 14 d rats gained less weight than C (Table 1) and showed a concomitant lower energy assumption and gWAT weight. After 5 h of food removal, no difference in glucose plasma levels was found.

**Figure 2:**
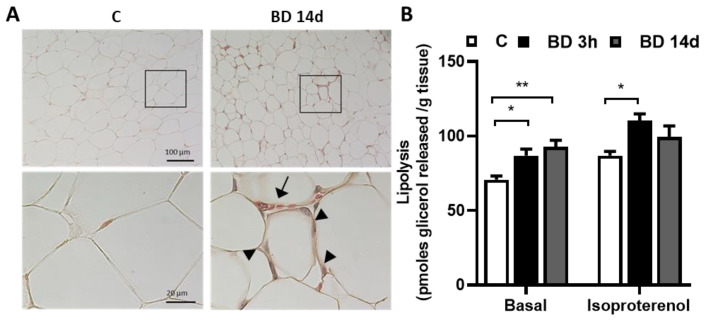
Effect of 14 days BD administration on gWAT morphology and lipolysis. (**A**) shows a representative histological analysis of gWAT from control (C) and BD-treated rats (BD 14 d). Below is the enlargement (100×) of the framed areas. Note the presence of capillaries (arrow) and infiltrating (inflammatory) cells/macrophages (arrowhead) around the adipocytes in BD 14 d-compared to C-gWAT. Hematoxylin and Eosin staining. (**B**) represents Basal and isoprotenerol-stimulated lipolysis. Values represent the mean ± ES of 8 different animals * *p* < 0.05, ** *p* < 0.01.

Morphological analysis indicates that 14 days of BD administration induces a reduction in adipocyte size (Figure 2); in addition, in gWAT, parenchyma macrophage infiltration and an enhancement in vascularization can be observed (Figure 2A). A reduction in tissue lipid accumulation has also been observed in the liver of BD 14 d group (Supplementary Figure S1).

BD induces a significant increase in basal lipolysis that was already evident three hours after the administration of their precursor. The observed increases were +23% and +31% in BD 3 h and BD 14 d vs. C, respectively. BD also stimulated isoproterenol-stimulated lipolysis, and the effect reached statistical significance only in the BD 3 h group vs. C (Figure 2B).

### 3.2. Effect of BD on Redox Homeostasis

**Figure 3:**
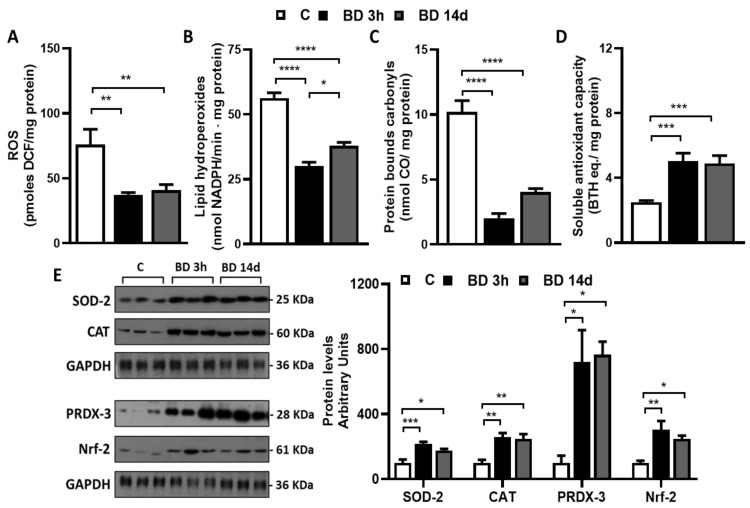
Effect of BD on gWAT redox homeostasis. Total ROS (**A**), lipid hydroperoxides (**B**), protein oxidative damage (**C**), and tissue soluble antioxidant capacity (**D**). Values are means ± SEM of 8 different rats. * *p* < 0.05, ** *p* < 0.01, *** *p* < 0.001, **** *p* < 0.0001. Enzymatic antioxidant capacity (**E**). Representative Western Blot of Superoxide dismutase-2 (SOD-2), catalase (CAT), peroxiredoxin-3 (PRDX-3), and Nuclear factor erythroid-related factor 2 (Nrf2) (**E**). GAPDH was used as loading control (25 μg of protein/rat/lane). Histograms represent the quantification of data. Data were normalized to the value obtained for control animals, set as 100. Values represent the mean ± SEM of 6 different rats or 3 rats in the case of PRDX-3.

To test if BD could affect gWAT redox homeostasis, we evaluated the tissue’s ROS levels, the oxidative damage to lipids and proteins, and the tissue’s antioxidant capacity (Figure 3).

BD was efficacious in reducing tissue ROS levels. The BD effect was observed already 3 h after its administration and persisted after 14 days of treatment. ROS levels were reduced by 52% and 46% in BD 3 h and BD 14 d vs. C, respectively. BD also protected proteins and lipids from oxidative damage. Indeed, when compared to C, gWAT lipid hydroperoxide levels were significantly reduced by 47% and 33% in BD 3 h and BD 14 d vs. C, respectively. Furthermore, carbonylated protein levels were strongly reduced in both BD 3 h (−80%) and BD 14 d (−60%) vs. C.

Concerning gWAT antioxidant capacity, we detected both soluble and enzymatic components by performing the ABTS assay and evaluating the protein levels of antioxidant enzymes. BD rapidly improved tissue soluble antioxidant capacity, which increased by 102% in the BD 3 h vs. C group, and this effect persisted after 14 days of treatment. It also enhanced SOD-2, PRDX-3, and CAT protein levels. The increases observed were +116% and +75% in BD 3 h and BD 14 d vs. C, respectively, in the case of SOD-2; +159% and +147% in BD 3 h and BD 14 d vs. C, respectively, in the case of CAT; and +619% and +666% in BD 3 h and BD 14 d vs. C, respectively, in the case of PRDX-3. The levels of Nrf-2, known as the main orchestrator of the cellular antioxidant response, were significantly and rapidly enhanced by BD, and the increases observed were +205% and 147% in BD 3 h and BD 14 d vs. C, respectively.

### 3.3. Effect of BD on gWAT ER Stress and Inflammation

**Figure 4:**
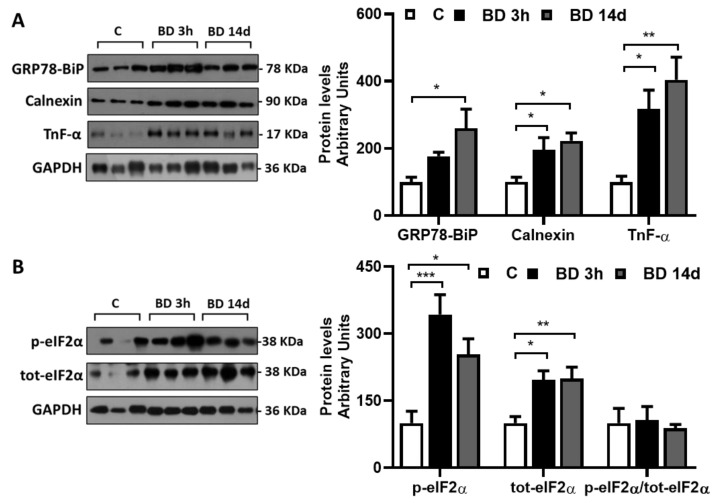
Effect of BD on gWAT levels of proteins linked to ER and cellular stress involved in ER stress and UPR^ER^ response detected. Representative Western blots of GRP78/BiP, Calnexin and TnF-α (**A**), and total and phosphorylated (ser 78) levels of eIF2α (**B**) were performed in gWAT lysate. GAPDH was used as loading control (25 μg of protein/rat/lane). Histograms represent the quantification of data. Data were normalized to the value obtained for control animals, set as 100. Values represent the mean ± SEM of 6 different rats. * *p* < 0.05, ** *p* < 0.01, *** *p* < 0.0001.

To evaluate the ability of BD to influence ER stress, we detected the protein levels of its specific markers, namely GRP78/BiP and calnexin (Figure 4A). Within 3 h after its administration, BD increased their protein levels, and the effect observed persisted after 14 days of treatment. GRP78/BiP levels were enhanced by +77% and +160% in BD 3 h and BD 14 d vs. C, respectively. The increases in calnexin levels were +96% and +123% in BD 3 h and BD 14 d vs. C, respectively.

BD enhanced both total and phosphorylated levels of eIF-2α (ser 51); the effect was evident within 3 h from its administration (Figure 4B). No significant changes in the ratio phosphorylated eIF-2α/total eIF2-α were observed between groups.

We also evaluated if BD could also lead to inflammation by evaluating TNF-α protein levels that were significantly higher in BD 3 h (+217%) and BD 14 d (+304%) vs. C.

### 3.4. Effect of BD on gWAT Oxidative Capacity and Mitochondrial Respiration Complexes Abundance

To test if BD could affect gWAT mitochondrial oxidative capacity, we detected tissue levels of mitochondrial respiratory complexes’ specific subunits, as an index of tissues complexes’ abundance, and Complex IV/cytochrome oxidase (COX) activity, as an index of the maximal mitochondria oxidative capacity.

**Figure 5:**
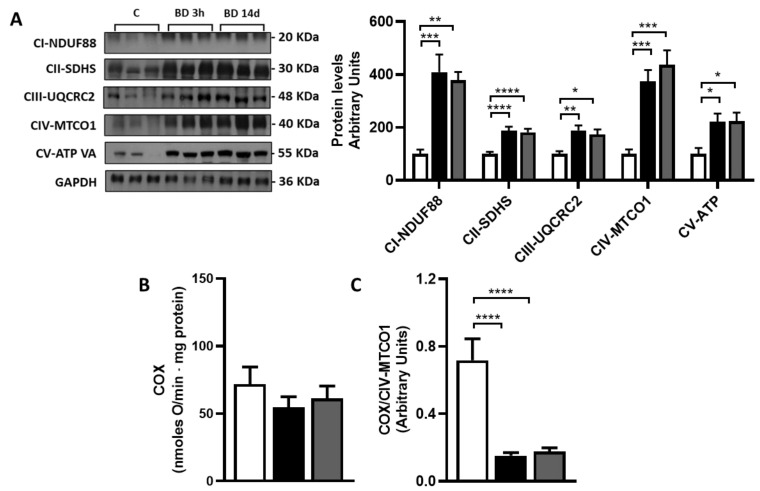
Effect of BD on gWAT mitochondrial respiratory complexes abundance and cytochrome oxidase activity. (**A**) shows representative Western Blots of specific subunits of the five mitochondrial respiratory complexes, namely CI-NDUF88, CII-SDHB, CIII-UQCRC2, CIV-MTCO1, and CV-ATP VA. GAPDH was used as loading control (25 μg of protein/rat/lane). Histograms represent the quantification of data. Data were normalized to the value obtained for control animals, set as 100. Values represent the mean ± SEM of 6 different rats. (**B**,**C**) show Cytochrome oxidase/complex IV activity reported as nmoles O/min mg tissue proteins (**B**) or normalized for Complex IV levels and expressed in arbitrary units (**C**). * *p* < 0.05, ** *p* < 0.01, *** *p* < 0,001, **** *p* < 0.0001. Values represent the mean ± SEM of 8 different rats.

BD increased all the respiratory complexes, the effect being rapidly manifested (Figure 5A). Concerning complex IV/cytochrome oxidase, the protein levels of its specific subunits MTCO1 were increased by about 300% in both BD 3 h and BD 14 d groups vs. C. Despite this, cytochrome oxidase activity, expressed as nmoles O/min mg proteins, was unaffected by BD at both treatment durations (Figure 5B). In addition, specific COX activity, obtained by normalizing tissue COX activity for the tissue amounts of complex IV, was significantly reduced in both BD 3 h and BD 14 d compared to C (−79% and −76%) (Figure 5C).

### 3.5. Administration of BD for 14 Days Affects Specific gWAT Adipokines/Cytokines Level

**Figure 6:**
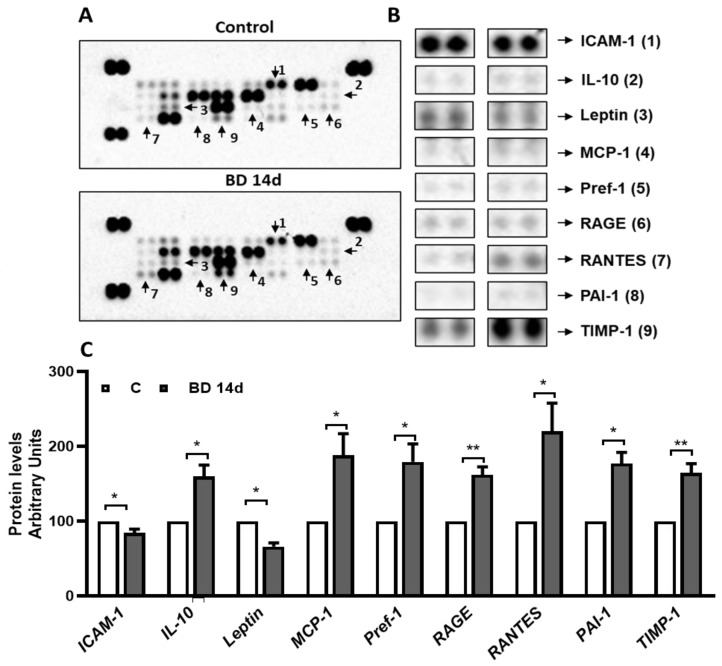
Effect of administration of BD for 14 days on gWAT adipokines profiles. Representative adipokine profile detected in gWAT from C and BD 14 d rats, by an adipokine protein array (**A**). The arrowheads indicate signals with significant changes. Magnifications of the spot of proteins whose intensity resulted statistically different are reported in (**B**). Histograms represent the quantification of relative levels of adipokines with observable changes (**C**). The values represent the mean ± SEM of 3 different samples, each one obtained by a pool of two different animals. Data were normalized to the value obtained for control animals, set as 100. * *p* < 0.05, ** *p* < 0.01.

Since adipokines/cytokines production could be affected by changes in gWAT mass and adipocytes size, we tested if the administration of BDs for 14 days could affect the adipokines levels in gWAT by performing an adipokine array. Representative blots were reported in Figure 6. When compared to C, the BD 14 d group showed an increase in chemotactic protein-1 (MCP1, +88%); Preadipocyte factor 1 (Pref-1, +79%); Advanced glycation endproduct receptor (RAGE, +62%); Regulated on activation, normal T cell expressed and secreted (RANTES, +120%); Plasminogen activator inhibitor-1 (PAI-1, +77%); TIMP metallopeptidase inhibitor 1 (TIMP-1, +64%); Interleukin-10 (IL10, +60%). In addition, downregulation of the adipokines Intercellular adhesion molecule-1 (ICAM-1) and Leptin was observed (−26% and −34%, respectively, vs. C).

## 4. Discussion

WAT is known to have a profound effect on overall energy metabolism, and a growing number of studies shed light on its paracrine and endocrine signaling function. Our study reports that BD greatly affects gWAT physiology and highlights the role played by its main product β-OHB on the tissue, whose effects are already evident within 3 h after the BD administration. The rapid effects induced by BD highlight that this molecule can act independently of its ability to reduce energy intake and determines long-term complex metabolic adaptations.

β-OHB plays a role in suppressing oxidative stress induced by oxidant treatment through increasing endogenous antioxidant capacities [5], and we report that BD enhances the whole tissue antioxidant soluble capacity, promotes the overexpression of proteins involved in enzymatic antioxidant capacity (SOD-2, PRDX-3, and CAT), and reduces tissue ROS content and the oxidative damages to lipids and proteins. Our data, showing the ability of BD to enhance SOD-2 and catalase levels in gWAT, agree with that reported in the literature indicating the effectiveness of β-OHB, in vitro added to 3T3-L1 adipocyte, to reduce ROS via augmentation of antioxidative stress factors [12]. Data also agree with the evidence that knockout mice for 3-Hydroxy-methyl-glytaryl-CoA synthetase 2, the first limiting enzyme in ketone bodies production, show a reduction in gWAT antioxidant enzymes [12]. The discovery that BD effects on the antioxidant system are already evident 3 h after BD administration extends the concept to the short-term action of β-OHB.

Multiple metabolic processes might be involved in the effect of BD to increase tissue soluble antioxidant capacity (for review, see Ref. [36]). Among these is the ability of β-OHB (i) to increase the mitochondrial Q/QH_2_ ratio and to decrease the NAD+/NADH ratio [37]; (ii) to decrease the glycolytic flux and force glucose-6-phosphate along the pentose phosphate pathway, thus leading to the production of two equivalents of NADPH; and (iii) to increase mitochondrial acetyl-CoA approximatively 15-fold, thereby increasing the flux of the citrate–pyruvate and citrate–isocitrate cycles, each of which includes a NADP+ to NADPH reduction step, catalyzed by malic enzyme and isocitrate dehydrogenase, respectively [38,39]. The β-OHB mediated increase in NADPH results in enhanced levels of reduced glutathione, thioredoxin, vitamins C and E, and other essential antioxidants [40]. Further detailed studies are needed to investigate if and how these pathways are enhanced in gWAT from BD-treated rats.

Among the transcriptional factors involved in the upregulation of antioxidant genes by the β-OHB in several tissues is Nrf2 [41]. We report that BD administration rapidly enhances Nrf2 protein levels in gWAT associated with increased levels of antioxidant enzymes content. However, the ability of β-OHB to activate gene transcription through inhibiting class I histone deacetylases (HDACs) could also be involved in the antioxidant effect of BD [5].

It is important to consider that a huge improvement of whole-tissue soluble antioxidant capacity, together with the overexpression of antioxidant enzymatic systems, leads to an excess of reducing equivalents that deplete ROS and drive the cells to reductive stress [42]. The change in redox homeostasis toward reductive stress profoundly affects ER functionality. In the ER, protein folding requires an oxidative environment; thus, the occurrence of reductive stress leads to the loss of protein disulfide bond formation and induces alteration in ER homeostasis (i.e., ER stress) and the activation of the UPR^ER^ [21]. These processes appear to manifest in the gWAT from BD-treated animals, as revealed by the potent antioxidant effect of BD described above, and by the rapid increase in levels of proteins involved in UPR^ER^, such as calnexin and BIP, that would counteract protein unfolding to limit ER stress.

One of the pathways of UPR^ER^ involves the phosphorylation of eIF-2α on ser 51. It is known that eIF2-α, together with GTP molecule and a charged initiator methionine tRNA, forms the active ternary complex that is fundamental for the translation initiation and ensures the correct identification of the start codon. P-eIF2α leads to a reprogramming of protein synthesis: the global protein synthesis is attenuated (thus reducing the influx of proteins into the ER), and the translation of a specific subset of mRNAs is enhanced through alternative non-AUG translation [43]. Here, we reported the ability of BD to enhance both P-eIF2α and total eIF2-α protein levels, with an unchanged ratio between them. Plausibly, the enhancement of total eIF2-α levels prevents the decrease in global protein synthesis that is generally associated with UPR^ER^, and it is possible to speculate its involvement in the rapid increase in specific protein levels induced by BD.

ER stress is strictly interrelated with mitochondrial dysfunction, and dysfunction on either side can compromise cell homeostasis and lead to metabolic derangements [22]. In addition, the occurrence of reductive stress has been reported to lead to mitochondrial functionality failure in adipose cells and myoblast [44,45]. In the present paper, we detected the activity of cytochrome oxidase/complex IV in gWAT homogenate, as an index of tissue mitochondrial maximal oxidative capacity, and we observed that BD administration did not affect it, despite being effective in increasing the tissue mitochondrial complex subunit IV levels, thus leading to a reduction in specific cytochrome oxidase activity (obtained by the ratio COX activity/Complex IV MCO1). These data could be indicative of the occurrence of mitochondrial dysfunction. However, further experiments concerning mitochondrial bioenergetic processes and mitochondrial ROS production are needed to clarify (i) whether changes in mitochondrial functionality occur in BD-treated rat samples and (ii) the relationship between the alteration of redox balance towards reductive stress and mitochondrial functionality in gWAT.

The occurrence of ER stress and mitochondrial dysfunction affects the ability of WAT to store lipids and tissue inflammation [17,46,47,48]. It has been reported that the induction of ER stress, both in vivo and in vitro, efficiently activated lipolysis in adipocytes [17,49]. In addition, in adipose tissue, ER stress leads to a pro-inflammatory state through the activation of pathways involved in the regulation of inflammatory cytokines release [23] (see also above). Furthermore, the proinflammatory cytokine TNF-α activates signaling pathways leading to adipocyte triglycerides breakdown [49,50]. Our data shed light on the ability of BD to stimulate lipolysis rapidly, being the effect plausibly mediated by the occurrence of ER stress, as well as by TNF-α. In the literature, the ability of β-OHB to inhibit lipolysis by binding to GPR109A receptors is also reported [7]. GPR109A shows EC50 for β-OHB of about 0.8 mM, which is similar to the concentration we found after 3 h from the administration of BD (0.94 mM) but higher than that observed following 14 days oral administration of BD in drinking water (0.46 mM). Thus, it is possible to speculate that in our experimental condition, the activation of lipolysis induced by ER stress and TNF-α prevails on its inhibition mediated by GPR109A.

Following 14 days of treatment with BD, we found a reduced accumulation of lipids in gWAT, as revealed by the lower tissue weight and the reduced adipocyte size. The above-interrelated processes leading to lipolysis (i.e., change in cell redox homeostasis, the occurrence of ER stress, reduction in mitochondrial oxidative capacity, inflammation), that manifest rapidly and persist after 14 days of BD treatment, could have contributed to the reduction of lipid accumulation in gWAT. However, data obtained in the BD 14 d group indicate that other processes could also have been involved; among these are changes in the level of adipokines involved in inflammation and adipocyte differentiation and the reduction in energy intake. It should be mentioned that BD taste may have contributed to influencing the appetite of the rats even if our data indicate that BD-treated rats drank an amount of solution similar to the water consumed by control rats, thus indicating that the treatment with BD was well tolerated by rats. Plausibly, the reduction of food intake could be due to the increase in β-OHB serum levels induced by BD. In fact, Langhans et al. [28,29] reported that a single subcutaneous administration of -β-HOB decreased food intake. Furthermore, Isoda et al. [51] reported the ability of BD to reduce food intake when administered by gavage to mice fed a high-fat diet, by sensitizing the action of leptin at the hypothalamic level.

By running an adipokines array, we reported that BD administration for 14 days induced changes in the levels of multiple adipokines/cytokines involved in inflammation, macrophages recruitment, and activation; among them were MCP1, RANTES, RAGE, and PAI-1, other than the TNFα cited above.

MCP1 affects adipocyte function, directs monocyte recruitment, and activates proinflammatory macrophages [23]. In line with this, in rats chronically treated with BD, associated with enhanced MCP1 levels, we observed macrophage infiltration into the gWAT (shown by histological analysis) and TNF-α enhanced levels. This observation is supported by the increased levels of RANTES, which participates in the recruitment of blood monocytes by triggering their adhesion and transmigration to/through adipose tissue endothelial cells [52], and of PAI-1, which play a role in the migration of macrophages and whose deletion or inhibition causes a significant reduction in M1 macrophages in the WAT of mice fed a high-fat diet [53].

Regarding RAGE, it is expressed at high levels in macrophages/monocytes, and the activation of this receptor by its ligands induces macrophage activation and mediates monocyte/macrophage chemotaxis and upregulation of inflammatory factors [54]. Thus, the upregulation of RAGE by BD treatment agrees with macrophage recruitment and inflammation. The evidence that BD also up-regulates the anti-inflammatory cytokine IL-10 suggests a limitation of the overall pro-inflammatory response. Plausibly, TNF-α itself could modulate IL10 upregulation by KBs, as suggested by studies reporting that IL-10 is up-regulated by TNF-a in vitro as well as in obesity both in humans and rodents [55].

Histological analysis indicates that 14 days of BD treatment induces an increase in capillaries surrounding adipocytes. The angiogenesis process is based on the fine regulation of protease/antiprotease balance; indeed, despite increased proteolysis being considered fundamental for endothelial cell migration and invasion, the inhibition of proteases plays an important permissive role during angiogenesis by preserving matrix integrity [56,57]. In line with this, after 14 days of treatment with BD, the vascularization of the tissue is enhanced, together with the levels of the serine protease inhibitor PAI-1 and the metalloprotease inhibitor TIMP-1. Some adipokines up-regulated by BD could contribute to the reduction of gWAT mass by inhibiting adipogenesis that, in adults, is essential for adipocyte turnover and maintenance of adipose tissue; among these are the already cited TNF-α [58], TIMP-1 [59], PAI-1 [60], as well as Pref-1 [61].

The data here reported also indicate that BD regulates WAT endocrine signaling. Leptin plays a crucial role in energy homeostasis by regulating the food intake and the oxidation rate of substrates [62]. Leptin production increases with increasing fat mass and adipocyte size. The reduction in leptin tissue production observed in BD-treated rats compared to control ones is consistent with their reduced fat mass and adipocyte diameter. On the other hand, these data contrast with the reduced energy intake observed in BD-treated animals, since notoriously leptin has an inhibitory effect on food intake, by activating hypothalamus POMC neurons and inhibiting the NPY one (recently reviewed in Obradovic et al., 2021 [63]). However, recent data have shown that the treatment with BD and the consequent increase in β-OHB levels increase the sensitivity of hypothalamic neurons involved in hunger circuitry and satiety to leptin [51], in line with our observations.

In this context, it should be underlined that the reduction in food intake observed in rats receiving BD administration for 14 days could have contributed to some of the effects here described. The evidence that most of the effects of BD-induced ketosis are observed within 3 h indicates that they are independent of the reduction in energy intake. However, further detailed studies will be necessary to define the real contribution of β-OHB per se and that due to the reduction of energy intake in such animals. Since the reduction in energy intake itself leads to an increase in KBs serum levels [62,64], the use of an animal model in which endogenous ketone body production is inhibited (i.e., 3-hydroxy-methyl-butyryl-CoA synthetase 2 knockout [12]) would be helpful to discriminate between the roles played by ketosis and energy intake reduction in the effects induced by long-term BD administration, and this is a limitation of our study.

## 5. Conclusions

Based on our data, we can suggest that BD treatment, by strongly enhancing the antioxidant capacity of the gWAT, shifts redox homeostasis toward reductive stress and, thus, to those interrelated consequences occurring at the level of ER and mitochondria that lead to lipolysis, tissue inflammation, and changes in gWAT adipokine/cytokines production.

In conclusion, by taking advantage of BD-induced increase in β-OHB levels, advances have been made in the understanding of the role played by KBs in influencing gWAT physiology. One of the most interesting and novel aspects reported in the present paper concerns the evidence that the effects induced by BD rapidly manifest and persist after 14 days of treatment. Studies based on a longer-term treatment with BD will allow to establish if these effects persist or if the occurrence of tissue adaptations leads to resolving ER stress and inflammation.

## Figures and Tables

**Table 1:** Effect of 14 days administration of BD to rat on body weight gain, energy intake, and gWAT weight.

	C	BD 14 d
Body weight at the beginning of the treatment (g)	273 ± 7	271 ± 4
Body weight at the end of the treatment (g)	329 ± 6	300 ± 7 **
Body weight gain (g)	55 ± 3	29 ± 4 ***
Energy intake (kJ)	3840 ± 363	2828 ± 348 ***
gWAT weight (g)	4.5 ± 0.4	3.2 ± 0.1 **
Glucose serum levels (mg/dL)	112 ± 6	120 ± 2

Values represent mean ± SE of 10 animals. ** *p* < 0.01, *** *p* < 0.001 vs. C.

## Data Availability

Data are contained within the article and Supplementary Material.

## Supplementary Materials

The following supporting information can be downloaded at: https://www.mdpi.com/article/10.3390/antiox12071471/s1, Figure S1: Effect of 14 days BD administration on liver morphology.
