# Supplementary material for: 1,3-Butanediol Administration Increases β-Hydroxybutyrate Plasma Levels and Affects Redox Homeostasis, Endoplasmic Reticulum Stress, and Adipokine Production in Rat Gonadal Adipose Tissue
Source: Antioxidants (Basel). 2023 Jul 22;12(7):1471. doi: 10.3390/antiox12071471 (PMC10376816; doi:10.3390/antiox12071471)
Supplement: Supplementary file 1 [file antioxidants-12-01471-s001.zip › antioxidants-2486983-supplementary.pdf]

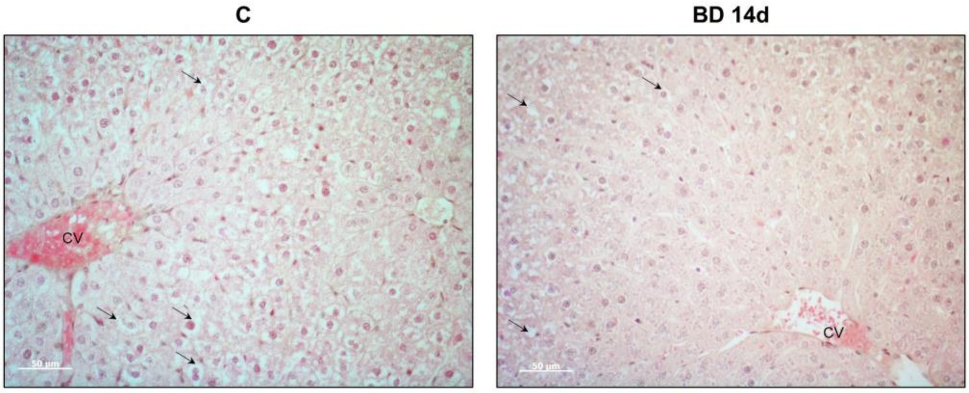

Figure S1. Effect of 14 days BD administration on liver morphology. Image shows a representative histological analysis of liver from control (C) and BD treated rats' administration (BD 14d). The analyses showed similar parenchymal structure and organization of hepatocytes. Note the lower presence of lipid depots (black arrows) in BD 14d group vs. control. No changes in vessels or central veins (CV) organization were detected. Hematoxylin and Eosin staining. Magnification used: 20X; scale bars applied: 50μm.
